# Supplementary material for: Determinants for late presentation of glaucoma among adult glaucomatous patients in University of Gondar Comprehensive Specialized Hospital. Case-control study
Source: PLoS One. 2022 Apr 29;17(4):e0267582. doi: 10.1371/journal.pone.0267582 (PMC9053799; doi:10.1371/journal.pone.0267582)
Supplement: S1 Questionnaire — (PDF) [file pone.0267582.s001.pdf]

Written Consent form:

Good morning/afternoon, my name is ------------------------------- I am a member of a research group working in Gondar university hospital tertiary eye care and training center. I have been studying determinants for late presentation of glaucoma using semi structured questionnaires and reviewing patient's medical record. Your own response for all of our questions are important to identify determinants for late presentation of glaucoma towards the study which is intended. Your response will be confidential and kept in secret. If you decide that you do not want to participate in the study now or at any time in the future, it is your right not to participate in the study. But we appreciate if you participate and will take 15-20 minutes for us to complete the questionnaire.

Can I have your permission to continue? YES □ NO □

If Yes, Continue

If No, Thank You!

S1 Questionnaire in English Language. Data collection tool for determinants and late presentation of glaucoma in English Language

S.N	Questions	Responses Category/answers	Remark
**I. Socio-demographic factors**			
1.1	Age at first diagnosis of glaucoma	_________________ (Years)	
1.2	Sex	1. Male<br>2. Female	
1.3	Educational status	1. No formal education<br>2. Primary<br>3. Secondary<br>4. College and above	
1.4	Occupation	1. Governmental employee<br>2. Non-governmental employee<br>3. Merchant<br>4. Farmer<br>5. Housewife<br>6. Others* (specifyy) ---------	
1.5	Monthly income (US$)	_________________	

1.6	How many kilometers is your residence from Gondar?	________________	
**II. Systemic disease related factors**			
2. Before you have been diagnosed with glaucoma			
2.1	Have you ever been diagnosed with diabetes?	1. Yes<br>2. No	
2.2	Have you ever been diagnosed with high blood pressure?	1. Yes<br>2. No	
2.3	Have you ever been diagnosed with Asthma?	1. Yes<br>2. No	
2.4	Have you ever had an accident/ injury to your eyes?	1. Yes<br>2. No	
**III. Knowledge related and Behavioral factors**			
3.1	Have you ever heard about the word glaucoma? (If no skip to 5.1)	1. Yes<br>2. No	

3.2	what is your source of information	1. Health professionals<br>2. Other people with glaucoma<br>3. Newspapers/Magazine<br>4. Television<br>5. Radio<br>6. Family member/friend<br>7. Other _______	
3.3	What is glaucoma?	It is high pressure in the eye<br>1. Yes<br>2. No<br>3. Not sure	
		It is a disease where nerve of the eye becomes weak<br>1. Yes<br>2. No<br>3. Not sure	

		It is damage to the nerve of the eye due to high pressure<br>1. Yes<br>2. No<br>3. Not sure	
		It is a blinding eye disease causing eye nerve damage<br>1. Yes<br>2. No<br>3. Not sure	
		It is an age-related process leading to decrease in peripheral vision<br>1. Yes<br>2. No<br>3. Not sure	
		It is an age-related process leading to decrease in vision<br>1. Yes<br>2. No<br>3. Not sure	
3.4		Increased Intra Ocular Pressure	

	What are the risk factors for glaucoma?	1. Yes<br>2. No<br>3. Not sure	
		Increasing age<br>1. Yes<br>2. No<br>3. Not sure	
		Family history of glaucoma<br>1. Yes<br>2. No<br>3. Not sure	
		Diabetes<br>1. Yes<br>2. No<br>3. Not sure	
		Smoking + alcohol using<br>1. Yes<br>2. No	

		3. Not sure	
3.5	Glaucoma can cause blindness?	1. Yes<br>2. No<br>3. Not sure	
3.6	Glaucoma can cause a permanents vision loss	1. Yes<br>2. No<br>3. Not sure	
3.7	Glaucoma has a hereditary pattern?	1. Yes<br>2. No<br>3. Not sure	
3.8	Glaucoma is due to spiritual problems?	1. Yes<br>2. No<br>3. Not sure	
3.9	Glaucoma can be managed with medications?	1. Yes<br>2. No<br>3. Not sure	
3.10	Glaucoma can be managed with surgery?	1. Yes<br>2. No<br>3. Not sure	
3.11	Glaucoma can affect all ages?	1. Yes<br>2. No<br>3. Not sure	

3.12	Is there anyone in your family who has glaucoma?	1. Yes<br>2. No<br>3. Not sure	
3.13	Do you regularly check-up your eyes every 2 years?	1. Yes<br>2. No	

IV. Ocular factors at the first diagnosis of glaucoma

	Ocular factors	OD	OS
4.1	Visual acuity		
4.2	Intra ocular pressure (mmHg)		
4.3	Presence of pseudo-exfoliation		
4.4	Vertical Cup to Disk ratio		
4.5	Type of glaucoma		
4.6	Ocular comorbidity (specify)		
