# Supplementary material for: Determinants for late presentation of glaucoma among adult glaucomatous patients in University of Gondar Comprehensive Specialized Hospital. Case-control study
Source: PLoS One. 2022 Apr 29;17(4):e0267582. doi: 10.1371/journal.pone.0267582 (PMC9053799; doi:10.1371/journal.pone.0267582)
Supplement: S2 Questionnaire — (PDF) [file pone.0267582.s002.pdf]

የፍቃድ መጠየቂያ ቅፅ:

ጤና ይስጥልኝ -------------- እባላለሁ ። የዩነቨርስቲው የጥናት ቡድን አባልነኝ ።

ግላኮማን (የዐይን ውሃ ግፊት) በተመለከተ ታካሚዎች በመጀመሪያ ወደ ጤና ተቋም ሲመጡ ያሉበትን ደረጃ እና ተያያዥ ምክንያቶች በጎንደር ዩኒቨርሲቲ ሆሰፒታል ህክምና እና ሳይንስ ኮሌጅ ለዓይን ህክምና የሚመጡ የግላኮማ (የዐይን ውሃ ግፊት) ታካሚዎች መካከል ቃለመጠይቅ በማድረግ እና የታካሚውን ቻርት በማየት እያጠናን እንገኛለን። ይህ ጥናት እርስዎ በሚሰጡን መረጃ ላይ የተመሰረተ ስለሆነ ፍቃድዎ ከሆነ መረጃውን በመስጠት ትብብር እንዲያደርጉልን በትህትና እንጠይቃለን ። በጥናቱ ላይ መሳተፍ የማይፈልጉ ከሆነ አሁንም ሆነ በሂደት ውስጥ አለመስማማት ይችላሉ ። ሆኖም ግን ጥናቱ ከትንሽ ጊዜ መፍጀት ውጪ ምንም አይነት ጉዳት የማያመጣ ስለሆነ እንዲሳተፉ እናበረታተለን ። መረጃዎ ምስጢራዊነቱ የተጠበቀ ፣ ለጥናቱ ብቻ የሚውል ና ለሌላ ጉዳይ የማንጠቀምበት መሆኑን ልናረጋግጥልዎ እንወዳለን። ቃለመጠይቁ 15-20 ደቂቃ የሚፈጅ ስለሆነ ፍቃደኝነትዎን በንግግርዎ እንዲያረጋግጡልን በትህትና እየጠየቅን ወደ ቃለመጠይቁ እንሄዳለን።

በጥናቱ ለመሳተፍ ፈቃደኛ ነዎት? ፈቃደኛ ነኝ □ ፈቃደኛ አይደለሁም □

S2 Questionnaire in Amharic (Local Language). Data collection tool for determinants and late presentation of glaucoma in Amharic language

ተ.ቁ	ጥያቄ	መልስ	ምርመራ
**የስነ-ሰብእ እና ማህበራዊ ጥያቄዎች**			
**1.1**	የታካሚው እድሜ ግላኮማ ሲገኝበት		
1.2	ጾታ	□ ወንድ □ ሴት	
1.3	የትምህርት ደረጃ	□ ማንበብ እና መጻፍ የማይችል<br>□ ማንብብ እና መፃፍ ብቻ<br>□ የመጀመሪያ ደረጃ ትምህርት ቤት<br>□ ሁለተኛ ደረጃ ትምህርት ቤት<br>□ ኮሌጅና ከዛ በላይ	
1.4	ሥራ	□ የመንግሥት<br>□ መንግሥታዊ ያልሆነ ድርጅት<br>□ ንግድ<br>□ ግብርና<br>□ የቀን ሰራተኛ<br>□ የቤት እመቤት<br>□ ጡረተኛ<br>□ ሌሎች ____________	
1.5	አማካይ ወርሃዊ ገቢ	በብር.................	

1.6	የመኖሪያ ቤትዎ ከሆስፒታሉ ያለው ርቀት	በኪ.ሜ....................	
**II. ከበሽታ ጋር የተዛመዱ ጥያቄዎች**			
2	ግላኮማ (የዐይን ውሃ ግፊት) እንዳለብዎ ከማወቅዎ በፊት፡		
2.1	በሃኪም የተረጋገጠ የስኳር በሽታ ነበረቦት?	አዎ □ አልነበረብኝም □	
2.2	የከፍተኛ የደም ግፊት ነበረቦት?	አዎ□ አልነበረብኝም □	
2.3	የአስም በሽተኛ ነበሩ?	አዎ □ አልነበርኩም □	
2.4	በዓይንዎ ላይ አደጋ ደረሶብዎት ያ ውቃል?	አዎ □ አያውቅምም □	
**III. ከእውቀት ጋር ተያያዥነት ያላቸው እና የሥነ ባህሪያዊ ምክንያቶች**			
3.1	ስለ ግላኮማ (የዐይን ውሃ ግፊት) ሰምተው ያውቃሉ?	አዎ□ አላውቅም□ (መልሱ አላውቅም ከሆነ ወደ ተ.ቁ 5.1 ይሂዱ)	
3.2	የመረጃ ምንጩ ምንድን ነው?	□ ከህክምና ባለሙያ<br>□ ከሌላ የግላኮማ ታካሚ<br>□ ከጋዜጣ/ከመፅሄት<br>□ ከቴሌቪዥን<br>□ ከሬድዮ<br>□ ከቤተሰብ/ከጓደኛ<br>□ሌላ ________________	

3.3	ግላኮማ (የዐይን ውሃ ግፊት) ምንድን ነው?		
	ከፍተኛ የዐይን ግፊት ነው	አዎ □ አይደለም □ አላውቅም□	
	የዐይንን ነርቭ የሚያደክም በሽታ ነው	አዎ □ አይደለም □ አላውቅም□	
	በከፍተኛ የዐይን ግፊት የዐይንን ነርቭ የሚጎዳ ነው	አዎ □ አይደለም □ አላውቅም□	
	ከእድሜ ጋር ተያይዞ የሚመጣ የዕይታ አድማስን የሚቀንስ በሽታ ነው	አዎ □ አይደለም □ አላውቅም□	
	ከእድሜ ጋር የተያይዞ የሚመጣ የዕይታ መጠንን የሚቀንስ በሽታ ነው	አዎ □ አይደለም □ አላውቅም□	
3.4	ለግላኮማ (ለየዐይን ውሃ ግፊት) አጋላጭ ሁኔታዎች ምንድን ናቸው		
	የየዐይን ግፊት መጨመር	አዎ□ አይደለም□ አላውቅም□	
	የእድሜ መጨመር	አዎ□ አይደለም□ አላውቅም□	
	በዘርሐረግ ከቤተሰብ	አዎ□ አይደለም□ አላውቅም□	
	የስኳር በሽታ	አዎ□ አይደለም□ አላውቅም□	
	ሲጋራ ማጨስ እና<br>አልኮል መጠጣት	አዎ□ አይደለም□ አላውቅም□	

3.5	ግላኮማ (የዐይን ውሃ ግፊት) ዐይነ ስውርነትን ሊያስከትል ይችላል	□ አዎ □ አይችልም □ አላውቅም	
3.6	ግላኮማ (የዐይን ውሃ ግፊት) ሊመለስ የማይችል የእይታ ማጣትን ሊያስከትል ይችላል	□ አዎ □ አይችልም □ አላውቅም	
3.7	ግላኮማ (የዐይን ውሃ ግፊት) በዘር ሊተላለፍ ይችላል	□ አዎ □ አይችልም □ አላውቅም	
3.8	ግላኮማ (የዐይን ውሃ ግፊት) በክፉ መንፈስ ሊመጣ ይችላል	□ አዎ □ አይችልም □ አላውቅም	
3.9	ግላኮማን (የዐይን ውሃ ግፊት) በመድኃኒት መቆጣጠር ይቻላል	አዎ □ አይችልም □ አላውቅም	
3.10	ግላኮማን (የዐይን ውሃ ግፊት) በቀዶጥገና መቆጣጠር ይቻላል	□ አዎ □ አይችልም □ አላውቅም	
3.11	ግላኮማ (የዐይን ውሃ ግፊት) ሁሉንም የእድሜ ክልል ሊያጠቃ ይችላል	□ አዎ □ አይችልም □ አላውቅም	
3.12	በቤተሰበ ውስጥ ግላኮማ (የዐይን ውሃ ግፊት) ያለበት አለ?	□ አዎ የለም □ አላውቅም	
3.13	አይንዎን በየጊዜው ይመረመራሉ?	አዎ□ አልመረመም□	
3,14	መልሰ አዎ ከሆነ በየስንት ጊዜ (በወር)	________	
