# Supplementary material for: Determinants for late presentation of glaucoma among adult glaucomatous patients in University of Gondar Comprehensive Specialized Hospital. Case-control study
Source: PLoS One. 2022 Apr 29;17(4):e0267582. doi: 10.1371/journal.pone.0267582 (PMC9053799; doi:10.1371/journal.pone.0267582)
Supplement: Attachment — Submitted filename: Response to Reviewers.docx [file pone.0267582.s004.docx]

# Authors’ response for the Editorials and reviewers’ comment

**Manuscript number:** PONE-D-21-27844

**Manuscript title**: Determinants for late presentation of glaucoma among adult glaucomatous patients in University of Gondar Comprehensive Specialized Hospital: Case-control study

# Responses to the Editorials

1. Please ensure that your manuscript meets PLOS ONE's style requirements, including those for file naming.

**Authors’ Response:** Thank you for your comment!

- *We accepted the comment,*
- *We checked and corrected based on the journal requirements point-by-point.*

2. We suggest you thoroughly copyedit your manuscript for language usage, spelling, and grammar. If you do not know anyone who can help you do this, you may wish to consider employing a professional scientific editing service. Upon resubmission, please provide the following: The name of the colleague or the details of the professional service that edited your manuscript. A copy of your manuscript showing your changes by either highlighting them or using track changes (uploaded as a *supporting information* file) A clean copy of the edited manuscript (uploaded as the new *manuscript* file)

**Authors’ Response:** Thank you for your suggestions!

- *We accepted the comment,*
- *All files were uploaded based on the requirement accordingly.*
- *All of the authors are participated and reviewed the manuscript for the language usage, spelling, and grammar.*
- *Specially, the authors listed below (The higher institutional employs who had “English language proficiency”) reviewed the manuscript in-depth.*
- *Mr. Natnael Lakachew Assefa (Assistant Professor of Optometry): Reviewed the document, responded the editors and reviewers comment, checked the language usage, spelling error, and grammar correction.*
- *Mr. Abel Sinshaw Assem (Lecturer of Clinical Optometry): Reviewed the manuscript, checked the language usage, spelling error, and grammar correction.*

3. Please include additional information regarding the survey or questionnaire used in the study and ensure that you have provided sufficient details that others could replicate the analyses. For instance, if you developed a questionnaire as part of this study and it is not under a copyright more restrictive than CC-BY, please include a copy, in both the original language and English, as Supporting Information

**Authors’ Response:** Thank you for your information!

- *We accepted the comment and submitted the data collection tool (****Questionnaire****) as supporting Information.*
- ***“S1 Questionnaire in English Language” and “S2 Questionnaire in Amharic (Local Language)”.***

4. Please provide additional details regarding participant consent. In the ethics statement in the Methods and online submission information, please ensure that you have specified whether consent was written or verbal/oral. If consent was verbal/oral, please specify: 1) whether the ethics committee approved the verbal/oral consent procedure, 2) why written consent could not be obtained, and 3) how verbal/oral consent was recorded. If your study included minors, please state whether you obtained consent from parents or guardians in these cases. If the need for consent was waived by the ethics committee, please include this information

**Authors’ Response:** Thank you for your comment!

- *We accepted the comment and described in detail*
- ***“Ethical requirement: Page 9, line 2-10” and “Consent form: S1, Page 1*”**

5. PLOS requires an ORCID iD for the corresponding author in Editorial Manager on papers submitted after December 6th, 2016. Please ensure that you have an ORCID iD and that it is validated in Editorial Manager.

**Authors’ Response:** Thank you for your comment!

- *We accepted the comment and authorization accessed with this link* [***https://orcid.org/0000-0002-4998-6128***](https://orcid.org/0000-0002-4998-6128)

6. In your Data Availability statement, you have not specified where the minimal data set underlying the results described in your manuscript can be found. PLOS defines a study's minimal data set as the underlying data used to reach the conclusions drawn in the manuscript and any additional data required to replicate the reported study findings in their entirety. **Authors’ Response:** Thank you for your comment!

- *We accepted the comment and uploaded sample data with this submission as a supporting Information:* ***“S3- Sample data”***

# Responses to the Reviewer #1

1. The results section of the abstract is not precise and they needs to be refocused.

**Authors’ Response:** Thank you for your comment!

- *We accepted the comment and revised the abstract section including the result section.*
- ***“Abstract result section: Page 2, line 13-17”***

2. The glaucoma diagnosis criteria should be clearly stated to provide context for the glaucoma diagnosis to provide a basis for comparison with other studies.

**Authors’ Response:** Thank you for your comment!

- *We accepted the comment and corrected.*
- ***“Methods section: page 6, line 18-20”:*** ***“Late glaucoma diagnosis:*** *Glaucomatous disc cupping of CDR > 0.8, in which there is no suggestion of other optic nerve pathology and typical glaucomatous field loss with a mean deviation of greater than -14 decibel in either of the* eyes.”

3. The classification of age is arbitrary i.e 18-40 then 41-50. Please use standard age classification such as 18-35 youth, 36-59 adult, 60 and older.

**Authors’ Response:** Thank you for your comment!

- *Age classification was done by SPSS software analysis based on the participant’s age range with interquartile method.*
- *The study participants included in our study were aged ≥ 18 years.*
- *Due to study subjects age variation and limited studies with same population characteristics in the study area, we classified based on statistics.*
- *If we used the suggested classification, most of the subjects would be in the same age group and difficult for analysis.*

4. Was only eye involved in the analysis where the glaucoma was bilateral? was there a correlation between the 2 eyes? if so then stick the use of one eye in the analysis.

**Authors’ Response:** Thank you for your comment!

- *All patients who fulfill the Late glaucoma diagnosis criteria of* ***CDR > 0.8*** *and* ***MD > -14 decibel*** ***in either eye*** *(Either* ***both eyes*** *or* ***one eye****) were included in the analysis.*
- ***“Described in Methods section: page 6, line 11-17”:***
- ***Cases*** ***(Late presenters):*** *Any chronic glaucoma patients diagnosed with cup to disc ratio (CDR) > 0.8, in which there is no suggestion of other optic nerve pathology and typical glaucomatous field loss with a mean deviation of greater than -14 decibel in either of the eyes at their first presentation [16, 18].*
- ***Controls:*** *Chronic glaucoma patients diagnosed with cup to disc ratio (CDR) < 0.8 and typical glaucomatous field loss with a mean deviation of < -14 decibel in both of the eyes at their first presentation [16, 18].*

5. How was Normal Tension Glaucoma diagnosed? Please explicitly outline

**Authors’ Response:** Thank you for your comment!

- *Since the diagnosis of all chronic glaucoma types were done by CDR and MD, Normal Tension Glaucoma was diagnosed with same criteria.*
- ***“Described in Methods section: page 6, line 18-20”: “Late glaucoma diagnosis:*** *Glaucomatous disc cupping of CDR > 0.8, in which there is no suggestion of other optic nerve pathology and typical glaucomatous field loss with a mean deviation of greater than -14 decibel in either of the eyes.”*

6. The table headings are to laborious. Authors should relook at them:

**Authors’ Response:** Thank you for your comment!

- *We accepted the comment and revised table headings:*
- ***“Result section: page 10, line 7-8; page 12, line 9-10; page 15, line 4-5”***

# Responses to the Reviewer #2

**Major comments:**

1. The experimental design is not rigorous and solid enough, also the results are not strong enough to support conclusion

**Authors’ Response:** Thank you for your comment!

- *We accepted the comment and we appreciated your suggestion:*
- *But the study design employed was cross-sectional not experimental:* ***“Methods section: page 5, line 3”.***
- *This study might have an inheritance limitation of the cross-sectional study design for the conclusion of cause and effects due to this we included this as a limitation of our study at the end of the discussion section:* ***“Discussion section: page 20, line 5-8”.***

2. Page 6. Methods: the definition of late presentation of glaucoma and controls are not clear, please add more detailed information about the selection of cases and controls (Please reference to Fraser S, Bunce C, Wormald R, Brunner E. Deprivation and late presentation of glaucoma: case-control study. BMJ. 2001 Mar 17;322(7287):639-43. doi: 10.1136/bmj.322.7287.639. PMID: 11250847; PMCID: PMC26542; Motlagh BF, Pirbazari TJ. Risk factors for late presentation of chronic glaucoma in an Iranian population. Oman J Ophthalmol. 2016 May-Aug;9(2):97-100. doi: 10.4103/0974-620X.184527. PMID: 27433036; PMCID: PMC4932803.).

**Authors’ Response:** Thank you for your comment!

- *We accepted the comment and revised the definitions of cases and controls based on your suggestion in the corrected manuscript operational definition section.*
- ***“Described in Methods section: page 6, line 11-17”:***
- ***Cases*** ***(Late presenters):*** *Any chronic glaucoma patients diagnosed with cup to disc ratio (CDR) > 0.8, in which there is no suggestion of other optic nerve pathology and typical glaucomatous field loss with a mean deviation of greater than -14 decibel in either of the eyes at their first presentation [16, 18].*
- ***Controls:*** *Chronic glaucoma patients diagnosed with cup to disc ratio (CDR) < 0.8 and typical glaucomatous field loss with a mean deviation of < -14 decibel in both of the eyes at their first presentation [16, 18].*

3. Page 10. Table 1. The classification of patients’ occupation is not logical enough. The standard occupational classification categorized into three groups: professional and technical occupations (I and II); manual and nonmanual skilled occupations (III and IV) and partly skilled and nonskilled occupations (V and VI). Please explain the reason about the classification in your manuscript.

**Authors’ Response:** Thank you for your comment!

- *We classified occupations based on the study participants’ occupational statues and considering the general population occupation classification of the study setting/area.*
- *Those suggested classification is not familiar classification in our settings.*
- *The study population occupations are categorized in one of the listed occupations in our study area,*
- *That was the reason and used these classifications to be consistent with other studies.*

**Miner comments:**

1. Page 3, introduction: “In 2013, the number of people aged 40 to 80 years with glaucoma was 64.3 million which was predicted to increase to 76.0 million in 2020 and 111.8 million in 2040”. Reference should be added.

**Authors’ Response:** Thank you for your comment!

- *We accepted the comment and reference is added in the corrected manuscript.*
- *“Tham YC, Li X, Wong TY, Quigley HA, Aung T, Cheng CY. Global prevalence of glaucoma and projections of glaucoma burden through 2040: a systematic review and meta-analysis. Ophthalmology 2014, 121(11):2081-2090.”*

2. Page 3, introduction: “In Africa and South Asia, the prevalence of undiagnosed glaucoma in the population has been reported to be more than 90%”. Please modify the sentence, current one is hard to understand.

**Authors’ Response:** Thank you for your comment!

- *We accepted the comment and rewritten the sentence clearly in the corrected manuscript.*
- ***“Introduction section: page 3, line 7-10”:*** *“Population-based studies in Asian countries showed a higher prevalence of glaucoma [3] and Primary Open Angle Glaucoma (POAG) is the most commonly reported [4-6]. The prevalence of previously undiagnosed glaucoma in South Africa was 87.0% [7].”*

3. Page 4, introduction: More information should be added to describe the significant of this study?

**Authors’ Response:** Thank you for your comment!

- *We accepted the comment and included more descriptive sentences for the significance of the study in the corrected manuscript.*
- ***“Introduction section: page 4, line 1-3 and line 7-9”***

4. Page 6: Methods: typo, it should be “systematic random sampling” but not “systemic random sampling”.

**Authors’ Response:** Thank you for your comment!

- *It was systematic random sampling method and described in the corrected manuscript methods section:*
- ***“Methods section: page 5, line 21-23 and page 6, line 1-6”:*** *“The study participants for both cases and controls were selected among glaucomatous patients on follow-up who visited the glaucoma clinic during the data collection period using systematic random sampling. The cases were recruited from late glaucoma presenters while the controls were selected among those without late glaucoma. The projected numbers in two months follow-up for cases and controls were 495 and 540 respectively. So, K_case_ = 495/246= 2.012 approximately 2 and K_control_ = 540/246 = 2.19 approximately 2. Both controls and cases were selected by systemic random sampling with a fraction of k=2 form their medical record numbers.”*

5. Page 6: Methods: Please provide more information about the questioner of knowledge-related factors in supplementary material.

**Authors’ Response:** Thank you for your comment!

- *We accepted the comment and provided the questionnaire as supplementary document in English language and Amharic (Local language|).*
- *“S1 Questionnaire in English Language and S2 Questionnaire in Amharic (Local Language)”*

6. Page 7: Methods: Definition about “Regular eye checkup”, dose it means eye checkup for every two years or only once in past two years? If so, it should not be defined as “regular” eye check-up but “frequency of ophthalmic examination in the last 2 years”.

**Authors’ Response:** Thank you for your comment!

- *We accepted the comment and corrected in the revised manuscript as “Those individuals who check up their eyes every two years”.*
- ***“Methods section: page 7, line 4-5”:*** ***“Regular eye checkup:*** *Those individuals who check up their eyes in every two years [17, 24].”*

7. Page 8: Methods: Software information (EPI Info, SPSS) should be provided, for example, the company name or website link.

**Authors’ Response:** Thank you for your comment!

- *We accepted the comment and indicted the company name in the revised manuscript.*
- ***“Methods section: page 8, line 9-10” :***
- *“EPI INFO Version 7:* [*https://www.cdc.gov/epiinfo*](https://www.cdc.gov/epiinfo)*”*
- *“SPSS Version 20:* [*https://www.ibm.com/analytics/spss-statistics-software*](https://www.ibm.com/analytics/spss-statistics-software)*”*

8. Page 10. Results, table 1. Are there any patients under 18 years old? And please clarify the unit for monthly income?

**Authors’ Response:** Thank you for your comment!

- *No: The study participants included in the study were adults aged equally or greater than 18 years which is described in the revised manuscript methods section.*
- ***“Methods section: page 5, line 9-10”:*** *All adult glaucomatous patients aged ≥, diagnosed within the last two years and on follow-up were included in the study.*
- *Unit monthly income was “US dollars (US$)” and corrected in the revised manuscript. “****Result section: page 11, table 1 and page 16, table 2”***

9. Page 15. Results, table 3. Monthly income in table 3 is not consistent with table1. Please keep it consistent in the whole manuscript.

**Authors’ Response:** Thank you for your comment!

- *We accepted the comment and corrected in the revised manuscript.*

10. Page 16. Results, table 3. Please define " POAG, CACG, PxG" in table 3 legend.

**Authors’ Response:** Thank you for your comment!

- *We accepted the comment and corrected in the revised manuscript.*

# Responses to the Reviewer #3

1. A main suggestion is to interpret the results in the public health context, i.e. to discuss what can be done from a public health perspective on timely glaucoma diagnosis based the observed results. The current Conclusion basically repeats the data results after a literature review, but what is really more important (and why the study was done in the first place) is how the study results and knowledge can inform potential interventions to prevent late glaucoma diagnosis in a typic resource limited setting in Africa, e.g. community based educational campaigns, information brochure in diabetes clinics etc.

**Authors’ Response:** Thank you for your comment!

- *We accepted the comment and suggestions:*
- *We added the significant of the study in the introduction section, conclusions were revised based on the objective of the study:* ***“Introduction section: page 4, line 1-3 and line 7-9”***
- *We revised the introduction section (the state of knowledge, significance of the study for the population and others):* ***“Introduction section: page 3, line 21-23 and page 4, line 1-6”.***
- *We revised the discussions and conclusions based on your comment and suggestions.*

2. Page 3, "In Africa and South Asia, the prevalence of undiagnosed glaucoma in the population has been reported to be more than 90%", this seems unbelievably high: >90% in the population have glaucoma. Glaucoma prevalence can't be this high.

**Authors’ Response:** Thank you for your comment!

- *We accepted the comment and revised the references and modified accordingly.*
- ***“Introduction section: page 3, line 7-10”:*** *“Population-based studies in Asian countries showed a higher prevalence of glaucoma [3] and Primary Open Angle Glaucoma (POAG) is the most commonly reported [4-6]. The prevalence of previously undiagnosed glaucoma in South Africa was 87.0% [7].”*

3. Page 11, the middle, "The median IOP of the over study participants was 25.80 mmHg and ...". Is this reporting the IOP at diagnosis time, or the IOP at time of this study's visit?

**Authors’ Response:** Thank you for your comment!

- *The data for IOP was taken from the patients’ medical record card at thier first diagnosis time not during the data collection time of the study.*
- ***Described in the manuscript methods section: page 7, line 22-23*** *and in the uploaded supporting information S1 and S2 data collection tool.*
