# Supplementary material for: Determinants for late presentation of glaucoma among adult glaucomatous patients in University of Gondar Comprehensive Specialized Hospital. Case-control study
Source: PLoS One. 2022 Apr 29;17(4):e0267582. doi: 10.1371/journal.pone.0267582 (PMC9053799; doi:10.1371/journal.pone.0267582)
Supplement: Attachment — Submitted filename: Response to Reviewers.docx [file pone.0267582.s005.docx]

# Authors’ response for the Editorials and reviewers’ comment

**Manuscript number:** PONE-D-21-27844

**Manuscript title**: Determinants for late presentation of glaucoma among adult glaucomatous patients in University of Gondar Comprehensive Specialized Hospital: Case-control study

# Responses to the Editorials

1. Journal Requirements:

   Please review your reference list to ensure that it is complete and correct. If you have cited papers that have been retracted, please include the rationale for doing so in the manuscript text, or remove these references and replace them with relevant current references. Any changes to the reference list should be mentioned in the rebuttal letter that accompanies your revised manuscript. If you need to cite a retracted article, indicate the article’s retracted status in the References list and also include a citation and full reference for the retraction notice.

**Authors’ Response:** Thank you for your comment!

- We checked each references and corrected based on the journal reference guidelines.
- We reviewed and checked the document point by point based on the comments and suggestions of the editor’s and reviewers’.

# Responses to the Reviewer #1

1. Reviewer #1: The correction indicated by the reviewer have been addressed and the now the paper reads more coherently and structurally conforms to the format of the journal.

**Authors’ Response:** Thank you in advance for your constructive comment and sharing your profound experience throughout the manuscript revision process.

# Responses to the Reviewer #4

1. Reviewer #4: This is an interesting study, although could be improved by a clearer argument for why previous literature from the area is limited and/or inappropriate, and so why this specific study is required. There are some grammatical slips throughout the manuscript, although these are minor. Specific comments are outlined, below.

**Authors’ Response:** Thank you in advance for your constructive comment and suggestions.

**Abstract**

1. two decimal places for age is probably excessive (similarly elsewhere in the manuscript)

**Authors’ Response:** we accepted and corrected with one decimal places in the new manuscript.

2. the abstract doesn’t give a definition of “late presenter”

**Authors’ Response:** we accepted and definition included in the new manuscript:

- **Page 2, line 7-9: *“****Late presenters were glaucoma patients diagnosed with cup to disc ratio (CDR) > 0.8 and mean deviation of greater than -14 decibel in either of the eyes at their first presentation.”*

3. “residing far away from the hospital” mention in the Conclusion is not listed in the results

**Authors’ Response:** These are resided > 53 km (the farthest km) away from the hospital which was described in:

- **Abstract results section: page 2, line 15:** *“resided > 53 km away from the hospital 6.02 times (AOR: 6.02; 2.76, 13.14”*
- **Results section: page 14, line 10-13: *“****Participants who resided 24 – 53 km and > 53 km away from UoG TECTC had the odds of 4.50 times (AOR: 4.50; 2.15, 9.40) and 6.02 times (AOR: 6.02; 2.76, 13.14) more likely being late presenter respectively compared to those who resided <3km away from the UoG TECTC.”*

**Introduction**

1. Pg 3, line 14: grammar

**Authors’ Response:** we accepted and rewritten in the new manuscript.

- **Page 3, line 13-14:** *“Several studies estimated that 10 – 33% of people with glaucoma were visually impaired at their first diagnosis [10-12].”*

2. The introduction could make a more clearly argued case for why the study is important: in particular, it cites three studies regarding what are the determinants of late presentation (including one from Africa), but then goes on to say “there is little published evidences [sic] for why some… have advanced disease at first diagnosis”.What are the gaps and/or conflicts in previous work that into this area, and how does the current study hope to address these? in particular, if the authors feel that the previous work from elsewhere in Africa is not applicable to Ethiopia, they should probably state so more explicitly: even better if they can cite supporting demographic / other evidence to support why this is the case. Some of the arguments made in the Discussion regarding weaknesses in previous studies from Africa (e.g. regarding sample size) could be mentioned explicitly in the Introduction.

**Authors’ Response:** we accepted and rewritten in the new manuscript.

- **Page 3, line 22-23 and page 4, line 1-6:** *“Glaucoma was one of the leading cause of irreversible blindness in Ethiopia, thus this study has an immense importance to salvage the community from glaucoma induced blindness. Ethiopian glaucoma patients have different socio-cultural, genetic, environmental and other population related factors compared to European, Asian and other African countries. Previous published evidences done in Ethiopia doesn’t explore the determinant factors for the severity of glaucoma at the first presentation. Thus, it needs explicit in situ study to identify the determinants for late presentation of glaucoma.”*

**Materials and Methods**

1. pg 5, Line 9: age criterion is missing

**Authors’ Response:** we accepted and *“****aged ≥ 18*** ***year****s”* is added in the new manuscript.

2. pg 6, line 21: questionnaire

**Authors’ Response:** we accepted and spelling is corrected in the new manuscript.

3. pg 7, line 15: “expertise”: experts?

**Authors’ Response:** we accepted and spelling is corrected as *“experts”* in the new manuscript.

4. pg 7, line 16: a bit more detail about the reliability check would be useful (assuming it involved retesting, how many people were retested, and what was the interval between testing?): or is this the information that is partly provided on page 8, line 1?

**Authors’ Response:** we accepted and corrected in the new manuscript.

- **Page 7, line 16-20:** *“The questionnaire was pre-tested for reliability and validity in 25 glaucomatous patients in another hospital (Bahir Dar Felege Hiwot referral eye Hospital) with the same methods and the content of the questionnaire was assessed for its clarity, completeness and modified accordingly. It was also checked for its reliability using a reliability test and has a Cronbach alpha value of 0.77.”*

**Results**

1. It is stated that a history of diabetes was significantly associated with late presentation: isn’t it the ABSENCE of a history of diabetes that is associated?

**Authors’ Response:** Having of history of diabetes was significantly associated, but ***“it is protective against late presentation***” and we described it in the corrected manuscript:

- **Result section: page 15, line 3-5:** *“On the other hand, those who had history of diabetes mellitus had 84% lesser odds of being late presenter (AOR = 0.16, 95% CI: 0.68, 0.38) compared to those who didn’t have diabetes.”*
- **We discussed this issues in discussion section: page 19, line 21-23 and page 20, line 1-4:** *“The present study also revealed that patients who had a history of diabetes mellitus are less likely to present late compared to those who didn’t have diabetes mellitus. The result is comparable with a study done in South Africa [18]. This might be due to the reason that diabetic patients are more likely to have regular medical and ocular examinations for diabetic retinopathy screening and follow-up, hence the opportunity to spot glaucoma at an earlier stage. This can be supported by the evidence of opportunistic detection of glaucomatous optic discs within a diabetic retinopathy screening [31].”*

**Discussion**

1. pg 18, line 20: grammar (sentence fragment)

**Authors’ Response:** We accepted and rewritten in the new manuscript.

- **Page 18, 20:** we corrected and rewritten the sentences.

2. pg 18, line 23: these cited reasons are rather generic: a much better job otargetingspecific reasons is done in the following paragraph when differences between the current study, and the previous South African study, are discussed.

**Authors’ Response:** We accepted and edited in the new manuscript: **Page 18, line 23.**
