# Supplementary material for: Determinants for late presentation of glaucoma among adult glaucomatous patients in University of Gondar Comprehensive Specialized Hospital. Case-control study
Source: PLoS One. 2022 Apr 29;17(4):e0267582. doi: 10.1371/journal.pone.0267582 (PMC9053799; doi:10.1371/journal.pone.0267582)
Supplement: Attachment — Submitted filename: Response to Reviewers.docx [file pone.0267582.s006.docx]

# Authors’ response for the Editorials and reviewers’ comment

**Manuscript number:** PONE-D-21-27844

**Manuscript title**: Determinants for late presentation of glaucoma among adult glaucomatous patients in University of Gondar Comprehensive Specialized Hospital: Case-control study

# Responses to the Editorials

1. Journal Requirements:
   Please review your reference list to ensure that it is complete and correct. If you have cited papers that have been retracted, please include the rationale for doing so in the manuscript text, or remove these references and replace them with relevant current references. Any changes to the reference list should be mentioned in the rebuttal letter that accompanies your revised manuscript. If you need to cite a retracted article, indicate the article’s retracted status in the References list and also include a citation and full reference for the retraction notice.

**Authors’ Response:** Thank you for your comment!

- We checked each references and corrected based on the journal reference guidelines.
- We revised the manuscript based on the comments and suggestions of the editor’s and reviewers’.

# Responses to the Reviewer #4

1. Reviewer #4: The manuscript has improved in several areas as a result of the authors’ revisions.

However, I find the revision to the Introduction disappointing; it is a superficial rewording, and hasn’t engaged with the criticisms made in my original review appended below:
“The introduction could make a more clearly argued case for why the study is important: in particular, it cites three studies regarding what are the determinants of late presentation (including one from Africa), but then goes on to say “there is little published evidences [sic] for why some… have advanced disease at first diagnosis”.

What are the gaps and/or conflicts in previous work that into this area, and how does the current study hope to address these? in particular, if the authors feel that the previous work from elsewhere in Africa is not applicable to Ethiopia, they should probably state so more explicitly: even better if they can cite supporting demographic / other evidence to support why this is the case. Some of the arguments made in the Discussion regarding weaknesses in previous studies from Africa (e.g. regarding sample size) could be mentioned explicitly in the Introduction.”

Several sentences cry out for appropriate references: “Glaucoma is one of the leading causes… [ref?]; Ethiopian patients have [various differences…] [ref?]; previous published evidences done in Ethiopia (sic).. [what evidence? references? Also, “evidence” is already plural - please correct].” At the moment, the modified paragraph is a list of unsupported assertions which weakens the paper substantially, particularly as it is this paragraph that is the one that is supposed to convince the reader there is a need for the current study.

**Authors’ Response:** Thank you very much for your constrictive comment and suggestions.

- We accepted your comment and revised each of the points you raised in the corrected manuscript.
- We revised the references and cited statements appropriately.
- We revised the significance of this study, the gaps of the previous studies and the justifications to conduct this research.
- We addressed the comments as follows:
- “……….for why some… have advanced disease at first diagnosis”

***Corrected in page 3, line 13-16:*** *“Several studies estimated that 10 – 33% of people* *with glaucoma had advanced disease and visually impaired at the first diagnosis due to their late presentation [10-12]. The reason for the late presentation was due to lack of early symptoms [13, 14], slowly progressive and asymptomatic of nature of glaucoma [15].*

- “What are the gaps and/or conflicts in previous work that into this area, and how does the current study hope to address these? ..............could be mentioned explicitly in the Introduction.”

***Corrected in page 4, line 2-10:*** “*Previous published evidences in Ethiopia didn’t have enough information to explore the determinant factors for the late presentation of glaucoma. Most of these studies were done to assess and the prevalence of blindness due to glaucoma [8, 9] and the associated factors of glaucoma [24]. Because of this, it needs explicit in situ study to identify the determinants for late presentation of glaucoma. In addition, the result of this study will provide base line information for the health care workers, researchers, health care planers, policy makers and other stakeholders accordingly.”*

- “Several sentences cry out for appropriate references”

***Corrected and cited appropriately, page 4, line 1 and 5:*** …..*Glaucoma was one of the leading cause of irreversible blindness in Ethiopia [8]……... Most of these studies were done to assess and the prevalence of blindness due to glaucoma [8, 9] and the associated factors of glaucoma [24].*
